# Wiki-Based Clinical Practice Guidelines for the Management of Adult Onset Sarcoma: A New Paradigm in Sarcoma Evidence

**DOI:** 10.1155/2015/614179

**Published:** 2015-02-16

**Authors:** S. J. Neuhaus, D. Thomas, J. Desai, C. Vuletich, J. von Dincklage, I. Olver

**Affiliations:** ^1^Australasian Sarcoma Study Group, Melbourne, VIC 3002, Australia; ^2^Cancer Council Australia, Sydney, NSW 2000, Australia

## Abstract

In 2013 Australia introduced Wiki-based Clinical Practice Guidelines for the Management of Adult Onset Sarcoma. These guidelines utilized a customized MediaWiki software application for guideline development and are the first evidence-based guidelines for clinical management of sarcoma. This paper presents our experience with developing and implementing web-based interactive guidelines and reviews some of the challenges and lessons from adopting an evidence-based (rather than consensus-based) approach to clinical sarcoma guidelines. Digital guidelines can be easily updated with new evidence, continuously reviewed and widely disseminated. They provide an accessible method of enabling clinicians and consumers to access evidence-based clinical practice recommendations and, as evidenced by over 2000 views in the first four months after release, with 49% of those visits being from countries outside of Australia. The lessons learned have relevance to other rare cancers in addition to the international sarcoma community.

## 1. Introduction

Sarcomas are rare malignant tumours of bone and soft tissue [1]. They include a heterogeneous group of malignancies and involve many anatomical sites and subtypes. There are approximately 850 new cases of sarcoma each year in Australia [2].

The rarity of sarcoma and its subtypes makes it challenging to determine optimal treatment strategies. Multidisciplinary input, including specialist pathology and radiology expertise, is essential to providing best clinical practice outcomes [3]. However, there are significant gaps in the evidence base used to underpin clinical decision making for patients with sarcoma and significant geographic and site-specific treatment disparities.

## 2. Why Clinical Practice Guidelines?

Existing clinical practice guidelines for the management of sarcoma, such as those released by European Society of Medical Oncology (ESMO), National Institute for Health and Clinical Excellence (NICE), and National Comprehensive Cancer Network (NCCN), are consensus-based guidelines [4–7]. As such, although a useful point of reference, these guidelines are not optimized to address a number of features of the Australian environment.

Such differences include marked geographic disparity in sarcoma management. For example, the probability of radiotherapy as a primary (presurgical) modality in Australia is largely determined by centre-based preferences and access to sarcoma specialist centres. Similarly, availability as well as involvement of paediatric oncology expertise in treating patients in the Adult and Young Adolescent (AYA) age range varies by referral centre and co-location of paediatric and adult treatment centres and/or local networks. In addition, the mixture of private and public health funding models in Australia and national approval processes and funding for drugs have implications for Australian practice guidelines. For example, trabectedin is approved and reimbursed for the treatment of sarcomas in Europe, but not Australia.

Low levels of evidence often underpin clinical sarcoma practice. Balanced with this is a pragmatic requirement for clinicians to make decisions on the optimal management of their individual patients. Treatment algorithms rely heavily on individual clinician experience and consensus of the multidisciplinary team. As a consequence, variance in care between clinicians and centres is common. Similarly, access to clinical trials varies by geography and centre and translational research integration is often opportunistic, rather than nationally coordinated.

Increasingly, new prognostic factors and therapeutic approaches for sarcoma are being identified. The rapidly expanding knowledge base, particularly in areas of targeted therapy and molecular genetics, along with changes to pathological coding, new imaging modalities, and advances in surgery and radiotherapy, makes keeping up to date with the latest development in sarcoma management a challenge for all involved [8].

## 3. Promote Consistency in Decision Making through Provision of Best Evidence

The primary aim of the Sarcoma guidelines process was to bring together lead clinicians managing sarcoma, across a range of disciplines, to develop common shared understanding of the current evidence and to identify key research gaps in an Australasian setting. By promoting consistency in decision making through provision of best evidence, the development of pathways of care, both state and national, was identified as natural sequelae to this process.

## 4. Problem with Printed Guidelines

Traditional printed clinical practice guidelines are resource intensive and become out of date almost as soon as released, by virtue of new evidence constantly being published [9].

Wide stakeholder engagement and consultation is important in any guidelines process but adds to the delay. Nonautomatic electronic searching of bibliographic databases makes literature searches time consuming and expensive. In addition, there is a paucity of evidence demonstrating the impact on practice of printed guidelines [10].

Confronted with these challenges, the small size of the Australian sarcoma community and the pragmatic reality of “time poor” clinical practitioners, an innovative solution was required.

The Australian sarcoma community was fortunate to be able to utilize a relatively new and modern methodology for guidelines developed by Cancer Council Australia.

## 5. Wiki Platform

The sarcoma guidelines project was a collaborative project between the Australasian Sarcoma Study Group (ASSG) and Cancer Council Australia (CCA) which commenced in 2011 and utilized Cancer Council Australia's Wiki platform.

The term Wiki is derived from “swift” in Hawaiian and is a web application that allows the creation and editing of interlinked web pages via a web browser using simplified markup language to facilitate online collaboration [11].

CCA has modified and customized the MediaWiki, an open source Wiki software application, to facilitate the guideline development process.

**Figure 1:**
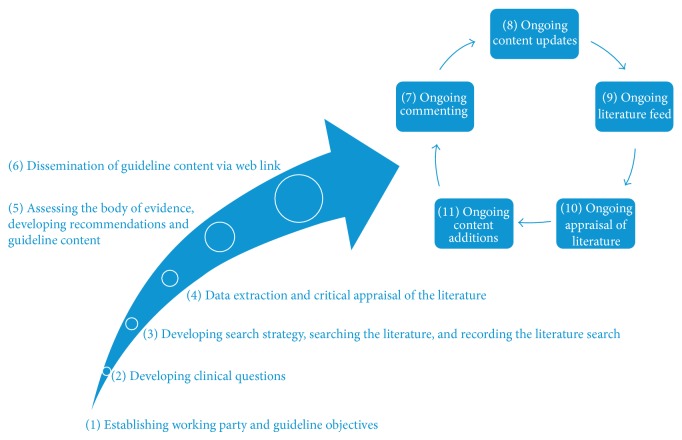
Developing guidelines on a Wiki platform.

The methodology and guideline development process has been translated into an online environment and adheres closely to that of traditional printed guidelines. The process is illustrated in Figure 1. The key difference is the ability to use the technological platform to provide continuous update of emerging literature as it becomes available [12]. In this way the Wiki sarcoma guidelines reflect the most recent, available, and up to date evidence base. CCA's Cancer Guidelines Wiki has access restrictions set in place, so that only authors can add content, but anyone can comment.

## 6. Methodology

A multidisciplinary working party including consumer representation was established in 2011. Expressions of interest were sought across the Australian sarcoma community, with the intent of providing both cross-discipline and geographic representation.

The working party comprised 42 members. The working party met at an initial “face to face” meeting to decide the clinical questions that were most relevant to their disciplines and determine the scope of the guidelines. The selected questions reflected the gaps in knowledge that impacted most on daily management decisions.

As an* ab initio* set of guidelines, the original scope of these guidelines was broad. The key areas covered have been refined to include

- diagnosis,
- multidisciplinary treatment,
- chemotherapy (systemic therapies),
- radiotherapy,
- surgery,
- follow-up.

Sarcomas affect children and adolescents, as well as adult members of the community. However, for reasons of pragmatism and resource, the scope of this first iteration is restricted to adult onset bone and soft tissue sarcoma. Gastrointestinal stromal tumours (GIST), Kaposi's sarcoma, and aggressive (desmoid) fibromatosis were excluded.

Childhood, adolescent (AYA), and gynaecological sarcomas are priorities for the next iteration of the guidelines.

Historically, clinical guidelines have been accompanied by a separate set of consumer guidelines. A decision was made not to do this with the clinical practice guidelines for adult onset sarcoma and reflects the availability of “online” consumer resources within Australia and the international community. The Wiki platform allows direct linkage to other relevant organisations' websites containing already available useful information for consumers. This has been integrated and linked to relevant sites from the guidelines table of contents page.

In addition, a number of external linkages have been embedded. These include links to the Australasian Sarcoma Study Group, geographic sarcoma specialist expertise, and multidisciplinary centres across Australia and linkage to available clinical trials sites such as the National Health and Medical Research Council (NHMRC) trials registry and sites presenting detailed treatment protocols such as EviQ.

## 7. Process

As with traditional printed guidelines, following definition of the clinical questions and standardised PICO (patient/population; intervention; comparison; outcome) formats were developed online. The next step involved literature search retrieval and assessment. The use of online literature search tools facilitated this process. Systematic search strategies were developed for a range of databases, such as PubMed, Embase, Trip database, and others using predetermined search term fields and predefined inclusion/exclusion criteria. Retrieved literature was made available via the Wiki platform as designated to individual working party authors and reviewers to assess using the online critical appraisal form, providing a body of evidence from which guideline content development was undertaken [11]. Manuscript appraisals are available as part of the audit trail of how the guidelines were derived and can be viewed online [13]. In addition, each author had to record conflict of interests, which can be viewed online, to provide transparency through the process.

The online nature of the Wiki provides a uniquely interactive format, where chapter authors and others can easily view all recommendations via the summary of recommendations page (illustrated at Table 1) and more detailed content of each of the clinical question pages from the guideline's table of contents or landing page.

**Figure 2:**
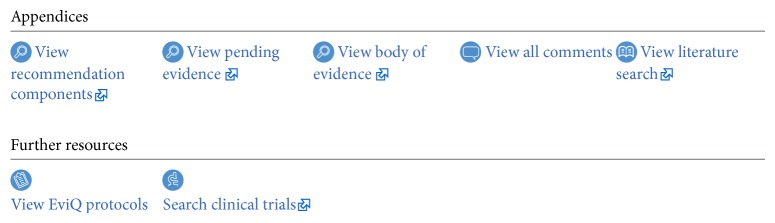
Appendices associated with each clinical question.

Each guideline question content page also contains the background methodological reports in the Appendices section. These Appendices, illustrated at Figure 2, provide transparency about the recommendation components, grading, and body of evidence used to generate the recommendation. Recommendation components include the grade, evidence base, evidence consistency, clinical impact, generalizability, and applicability to the Australian environment. In addition, users can view pending evidence, which has not yet been either assessed or incorporated into the body of evidence demonstrating at a glance the guideline's currency and allowing direct access via link to citations and abstracts. Users can also access individual critical appraisals of literature by navigating to the respective citation page.

The draft Clinical Practice Guidelines for Management of Adult Onset Sarcoma containing 54 recommendations and 35 practice points were released for initial public consultation for a 30-day period on 3 September 2013. The consultation process involved soliciting public comments by sending email alerts to recipients comprising relevant professional organisations, state and territory Cancer Councils, and individual clinical experts and consumer organisations in Australia and New Zealand. Organisations and individuals were invited to post comments on the Cancer Council Australia Cancer Guidelines Wiki. During the public consultation phase nine public comments (by five submitters) were received. The site received 488 visits (72% from Australia, 4% from New Zealand, 3% from United States, and remaining 21% from 37 other countries). These led to further edits, which were reviewed in detail by the working party.

The guidelines were released nationally on 15 November 2013 and can be accessed at http://wiki.cancer.org.au/australia/Guidelines:Sarcoma. From release in November 2013 to May 15, 2014, the guidelines received 3,475 page views with 1,344 visits and an average of 2.6 pages per visit. Of these 52% of visits were from Australia, 8.9% from USA, 6% from United Kingdom, 6% from India, 2% from Singapore, 1.9% from Germany, and the remaining 25.3% from 39 additional countries.

The guidelines highlight the importance of early referral to multidisciplinary centers that specialize in treating sarcoma. Caseload and experience is associated with improved rates of functional limb preservation, lower rates of local recurrence, good rates of overall survival, and improved quality of life. These centers are usually involved in ongoing clinical trials, in which sarcoma patients' enrollment is highly encouraged.

The importance of the multidisciplinary team in initial assessment, diagnosis, and making decisions about treatment is strongly endorsed by the recommendations in the guidelines. A multidisciplinary approach (involving pathologists, radiologists, surgeons, radiation therapists, medical oncologists, and paediatric oncologists, with experience in sarcoma), or within reference networks sharing expertise and treating a high number of patients annually, is preferred.

It should be noted that participants in this guideline development process found challenges in assigning conventional levels of evidence for many recommendations. The heterogeneity and rarity of sarcomas, along with the increased molecular stratification of clinical trials, means that few studies reached what would be considered the “gold standard” in other diseases (multiple placebo-controlled double-blinded randomized controlled trials), including more common cancers. The absence of evidence, however, does not exonerate clinicians from the necessity to make clinical judgments in caring for patients with sarcomas. This raises questions about the need to develop standards of evidence that recognize the challenges of research in rare diseases, including sarcoma. Consequently these guidelines emphasise the need for increased participation in collaborative research and trials programs as a standard of care.

## 8. Next Steps

The Wiki platform provides the sarcoma guidelines with an iterative and constantly updating framework. Infrastructure is in place to automatically feed literature updates from PubMed and Embase to relevant question authors [14]. In addition, new or emerging evidence can be manually submitted by the experts at any time using the commenting and submit new evidence features embedded within each question page. The ability to appraise supporting evidence for new therapies in a timely manner is particularly important in Australia, to potentially decrease the lag time before these therapies, which may be available internationally, and can be brought into national clinical practice. Guidelines also promote evidence-based recommendations which may be helpful in lobbying for the funding of a new drug.

Expert working party authors will continue at regular intervals to assess new evidence and comments and update the content where necessary, ensuring that this remains an iterative and current guideline. We will work towards empowering small writing groups, to whom we will provide new literature as it is published, with the ability to update their section of the guidelines without requiring specific public consultation. While reliant on small working groups, any bias is mitigated by the design of the platform where the public and experts can post comments at any time. In addition to this, the larger working group will meet annually to review all updates.

The working party will meet in November 2014 as part of an annual process to review the content and updates and formulate the next generation of clinical questions (Paediatric/AYA). Gynaecological sarcoma questions will be incorporated in 2016.

Defining the “research gaps” in sarcoma care has been an important outcome of this process. In each section these are included in the form of future research questions that need to be addressed by good quality collaborative trials.

As a strategy for boosting implementation, Cancer Council Australia is developing educational modules to accompany each of the online guidelines established. This process will be managed by adopting spaced education (called Qstream) techniques for online education. Qstream utilises clinical scenarios presented in a short answer test question format [15]. Further information is provided in response to answers and wrong answers, thus presenting more data iteratively over several weeks. Experience with spaced education modules has been shown to increase knowledge and retention of guideline content and change clinical practices [16].

Education modules can be linked to key stakeholder groups such a radiology, pathology, primary care provider, and surgical colleges. Such web-based education resources provide a cost-effective way of engaging with health providers and consumers who may not otherwise have been able to access these opportunities and can be stratified by level of expertise (e.g., medical student versus specialist) and by resource availability.

These guidelines are intended to be a resource to help create awareness, for use in medical education and as a resource in multidisciplinary team meetings. Engagement from the breadth of the sarcoma community, both in Australia and internationally, via comments and submissions of new evidence, is actively encouraged [17].

Australia is situated within a region containing the largest growing population in the world: currently over 4.3 billion [18]. There is significant economic and resource disparity across the Asia-Pacific region, with rapid growth in socioeconomic status and rising expectations in health care for large countries such as India and China [19]. However, emerging Internet technology provides a cost-effective way to address engagement and education and engage with and connect previously isolated research teams and extend expertise and clinical collaboration across the region in new and innovative ways. Due to the online nature of the guidelines we can easily add new or emerging questions of relevance. Similarly, we can retire questions that become irrelevant for clinical practice.

We hope these guidelines will provide an accessible up-to-date platform for dissemination of current evidence in a rapidly changing landscape and assist in clinical management of adult onset sarcoma. In addition, the guidelines provide a national and regional resource for multidisciplinary sarcoma teams, individual clinicians, students, and consumers. Cancer Council Australia are developing a suite of Wiki based guidelines for other cancers including melanoma and lung cancer. Sharing our methodology with international guideline developers has allowed the sharing of literature searches. Such international collaboration and sharing is key to expanding the reach of the guidelines.

## 9. Conclusions

Advances in multidisciplinary care have improved the evaluation and care of patients with sarcoma. One of the key principles underlying the development of these guidelines was to address issues where the evidence was unclear, where divergent interpretations of evidence existed, or where particular issues unique to the Australian setting needed to be considered.

The Australian Clinical Practice Guidelines for the Management of Adult Onset Sarcoma are the first step towards more standardised care for patients with sarcoma across the nation and provides a framework to educate the community about referral pathways and develop more formal communications between sarcoma centres and clinicians, particularly in relation to current trials and access.

The Cancer Council Australia Cancer Guidelines Wiki platform used to develop these guidelines is unique. It enables iterative, ongoing, and interactive guideline development and revision processes and provides a cost-effective, efficient methodology and transparent assessment of the available evidence in sarcoma management. There are significant opportunities to leverage this platform for further international engagement and collaboration.

## Figures and Tables

**(a):** 

Does referral to a specialist centre improve outcomes?	
+ Recommendation	Grade

Patients with suspected sarcoma to be referred to a specialist sarcoma unit prior to diagnosis in order to reduce the rates of incomplete excision, reoperation, and local recurrence and to improve survival.	C

**(b):** 

Chemotherapy (systemic therapies)	
What is the role for adjuvant systemic therapy for adults with BSTT?	
+ Recommendation	Grade

Curative treatment of Ewing's sarcoma comprises a combination of chemotherapy and surgery and/or radiotherapy.	B
The use of postoperative chemotherapy in adult type soft tissue sarcomas is not the current standard of care.	D
Curative treatment of high-grade osteosarcoma comprises chemotherapy and surgery.	B

## Key Message

Australian Clinical Practice Guidelines for the Management of Adult Onset Sarcoma were released in 2013. They constitute evidence-based guidelines in an accessible, interactive, and transparent format, which can be continuously reviewed and widely disseminated as new evidence becomes available.
